# Effectiveness and Personalized Approaches in the Correction of Gummy Smile: A Systematic Review of Orthodontic and Surgical Treatments

**DOI:** 10.3390/jcm13226843

**Published:** 2024-11-14

**Authors:** Alessio Danilo Inchingolo, Angelo Michele Inchingolo, Fabio Viapiano, Anna Netti, Anna Maria Ciocia, Irene Ferrara, Antonio Mancini, Andrea Palermo, Francesco Inchingolo, Gianna Dipalma

**Affiliations:** 1 Department of Interdisciplinary Medicine, School of Medicine, University of Bari “Aldo Moro”, 70124 Bari, Italy; ad.inchingolo@libero.it (A.D.I.); angeloinchingolo@gmail.com (A.M.I.); viapianofabio96@gmail.com (F.V.); annanetti@inwind.it (A.N.); anna.ciocia1@gmail.com (A.M.C.); ire.ferra3@gmail.com (I.F.); dr.antonio.mancini@gmail.com (A.M.); giannadipalma@tiscali.it (G.D.); 2 College of Medicine and Dentistry, Birmingham B4 6BN, UK; andrea.palermo2004@libero.it

**Keywords:** gummy smile, gingival display, orthodontic, skeletal anchorage devices, vertical maxillary excess, aesthetics

## Abstract

**Background/Objectives:** This systematic review seeks to assess the effectiveness of different orthodontic and combined orthodontic–surgical approaches for correcting gummy smile, with a focus on treatment efficacy, duration, and the potential for integrating various techniques. The objective is to offer evidence-based recommendations for the optimal management of gummy smile. **Methods:** A thorough search of the literature was conducted in the PubMed, Cochrane Library, Scopus, and Web of Science databases, covering publications from 1 January 1982 to 4 November 2024. Only randomized controlled trials involving adult human subjects with available full-text articles were included, while systematic reviews, editorials, case reports, and studies involving animals or in vitro experiments were excluded. Studies were selected based on their relevance to orthodontic or combined orthodontic–surgical treatments for correcting excessive gingival display (gummy smile), particularly in cases where residual growth does not impact treatment outcomes. The primary focus was on evaluating the efficacy of these interventions in improving smile aesthetics. Follow-up data were considered when available, though not required for inclusion. **Results:** The findings revealed that both orthodontic and surgical methods are effective in reducing gingival display. Orthognathic surgery offers lasting outcomes, particularly for patients with vertical maxillary excess, whereas orthodontic treatments, including the use of skeletal anchorage devices, are particularly effective for less severe cases. Minimally invasive approaches, such as botulinum toxin injections, provided temporary but promising results for patients reluctant to undergo surgery. **Conclusions:** Orthodontic and surgical techniques are viable options for treating gummy smile, with treatment choices depending on the condition’s severity. Future research, particularly long-term randomized studies, is required to further refine treatment protocols and improve patient outcomes.

## 1. Introduction

Gummy smile, or excessive gingival display (EGD), is defined as the exposure of more than 2–3 mm of gingival tissue when smiling [1,2]. The aesthetic threshold for considering gingival exposure “excessive” typically starts at 3 mm, though the perception of this can vary depending on patient and cultural factors. Studies suggest that an optimal smile exposes approximately 1–2 mm of gingiva, while anything above 3 mm is generally considered aesthetically undesirable [3,4]. The condition occurs in approximately 10% to 30% of the general population, and while it may not always be perceived as problematic in certain cultures, for many patients, it represents a significant source of aesthetic concern and psychological distress. The etiology of a gummy smile is multifactorial and may involve skeletal, dental, or muscular components or a combination of these factors [5,6].

**Figure 1:**
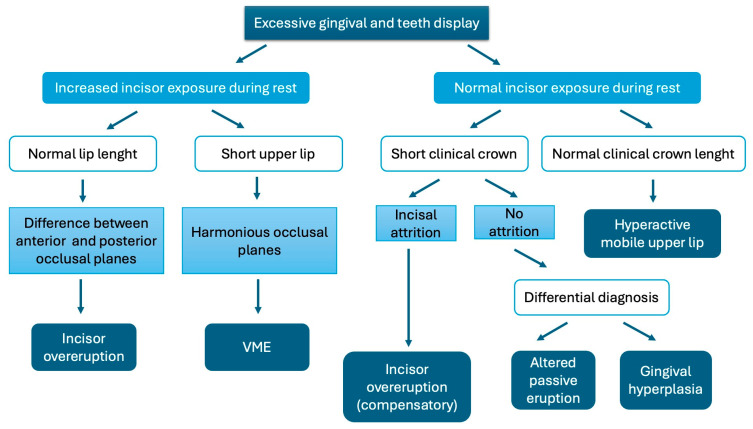
Diagnostic Flowchart for Gummy Smile.

From a diagnostic perspective, it is essential to understand the specific etiology of each case to select the most appropriate treatment (Figure 1).

Vertical maxillary excess (VME), a skeletal cause, is one of the most common contributors and involves an overgrowth of the maxillary bone [7]. This results in an elongated midface, which pushes the upper dentition and gingival tissues downward, leading to excessive gingival display [8]. Patients with VME typically exhibit more than 5–7 mm of gingival exposure, with the severity of VME classified into three categories: mild (2–4 mm); moderate (4–8 mm); and severe (>8 mm) [2]. The severity of VME directly influences the choice of treatment, often necessitating a surgical approach in moderate to severe cases [9,10].

Altered passive eruption (APE) is another dental etiology where the gingival tissues fail to recede fully during tooth eruption, leaving an excessive amount of gingiva covering the crowns of the teeth [11]. This condition often results in short clinical crowns, which can be objectively measured using the crown-to-root ratio [12]. A typical maxillary central incisor has a crown height of 10–12 mm, but in APE cases, this can be reduced by 2–3 mm or more due to excess gingiva [13]. Accurate diagnosis of APE requires clinical probing to assess the gingival margin relative to the cementoenamel junction (CEJ) [14]. If the gingival margin is more than 2 mm coronal to the CEJ, APE is likely present. Radiographic analysis can also confirm if the osseous crest is positioned too close to the CEJ, further complicating the eruption process [15,16].

Muscular factors, particularly hyperactivity of the upper lip elevator muscles, contribute to what is commonly termed a “dynamic” gummy smile. In these cases, the muscles, including the levator labii superioris, levator anguli oris, and zygomaticus minor, contract excessively during smiling, elevating the upper lip more than usual [17]. The average lip elevation during a smile is 6–8 mm, but in patients with hyperactive muscles, this elevation can exceed 10 mm, resulting in exaggerated gingival exposure. In such cases, muscular modification techniques, such as botulinum toxin injections, may be considered [18,19,20].

The diagnosis begins with an evaluation of the patient’s facial proportions, particularly the lower third of the face, which is critical in diagnosing vertical maxillary excess. The face is divided into three equal horizontal thirds, with the lower third encompassing the chin to the base of the nose. In cases of VME, the lower third is often elongated, which can be objectively measured using cephalometric analysis. Cephalometric values such as the mandibular plane angle (MPA) and anterior facial height (AFH) are increased in patients with VME. Typical cephalometric measurements for a harmonious smile include an AFH-to-total facial height ratio of approximately 55–57% [21,22].

An intraoral examination should assess the clinical crown height of the maxillary anterior teeth. In normal conditions, the central incisors should have an exposed crown height of 10–12 mm, as mentioned earlier. Measurements of less than this, coupled with excessive gingival coverage, may indicate altered passive eruption. Additionally, evaluating the gum line relative to the CEJ using probing techniques is critical. Measurements beyond 2 mm from the CEJ to the gingival margin suggest the presence of altered passive eruption [23,24,25].

Lip dynamics should be evaluated by observing the degree of upper lip elevation during smiling. Patients with hyperactive upper lip muscles typically show more than 10 mm of gingival exposure due to exaggerated lip movement. This can be objectively assessed using video capture or digital smile analysis software, which allows for precise measurement of lip movement in real time. Electromyography (EMG) can also be used in research settings to quantify muscle hyperactivity, though it is not typically necessary for routine clinical diagnosis [26,27].

Panoramic or cone-beam computed tomography (CBCT) imaging can be used to assess the skeletal and dental structures in more detail. Cephalometric analysis is particularly important for identifying skeletal abnormalities such as vertical maxillary excess. Key cephalometric indicators include an increased Sella–Nasion to Maxillary Plane angle (S–N/MP angle) and anterior maxillary height (AMH). Normal S–N/MP values range from 32° to 36°, but in cases of VME, these values are often significantly higher, correlating with the increased facial height [28,29].

The clinical implications of gummy smile extend beyond aesthetics. Numerous studies have demonstrated the negative psychosocial impact of excessive gingival display on patients, contributing to reduced self-esteem and social withdrawal. These patients often report avoiding smiling in public or during social interactions. It is critical to approach gummy smile treatment not only from an aesthetic standpoint but also from a psychological perspective. Addressing the patient’s concerns and providing a treatment plan that improves both their appearance and self-confidence is essential for achieving a successful outcome [30,31,32].

Orthodontic treatments are highly effective for dental causes, such as altered passive eruption or excessive eruption of the maxillary anterior teeth [33,34]. Techniques like fixed appliances and TADs (temporary anchorage devices) can reposition teeth and reduce gingival exposure [35,36,37]. TADs, in particular, offer stable anchorage, allowing for precise control of tooth movement [38,39]. Orthodontic treatment typically spans 12 to 24 months, with longer durations required for more complex cases [40,41].

For skeletal causes, such as vertical maxillary excess, orthognathic surgery is often the treatment of choice [42,43]. This surgical procedure repositions the maxilla to reduce gingival display and correct any associated functional issues, such as malocclusion [36,44]. While orthognathic surgery is invasive, it offers long-lasting and predictable results, particularly when combined with pre- and post-surgical orthodontics [45,46,47].

In cases of muscular hyperactivity, minimally invasive treatments like botulinum toxin injections provide a temporary solution [48,49,50]. By reducing the activity of the upper lip elevator muscles, botulinum toxin can limit gingival exposure during smiling [51,52]. However, these effects are temporary, typically lasting 3–6 months, necessitating repeated treatments for sustained results [53,54].

Recent advancements in diagnostic tools and treatment modalities have allowed for a more tailored approach to the management of gummy smile [55,56,57]. Three-dimensional imaging techniques, such as cone-beam computed tomography (CBCT), coupled with digital smile analysis software, have revolutionized the assessment of skeletal and soft tissue relationships [58,59,60]. These technologies enable precise measurements of the facial skeleton, tooth positioning, and soft tissue dynamics, facilitating a more accurate diagnosis and customized treatment plan. Moreover, the integration of minimally invasive approaches, such as laser-assisted gingivectomy or soft tissue contouring, has expanded the therapeutic options available to patients with mild to moderate gummy smile. These procedures, often used in conjunction with orthodontic techniques or botulinum toxin therapy, offer promising outcomes with reduced recovery times and minimal discomfort. The continuous evolution of these tools and methods highlights the importance of a multidisciplinary approach in the comprehensive management of gummy smile, emphasizing the need for further clinical research to refine treatment protocols and improve patient outcomes [61,62,63].

The primary objective of this review is to examine in detail the orthodontic and orthodontic–surgical techniques for the correction of gummy smile, with particular attention to efficacy, duration, and the combination of treatments. This review aims to provide a comprehensive overview of the available options and offer evidence-based recommendations for effective clinical management [64,65,66,67].

This review contributes uniquely to the field by addressing a critical gap in the literature, offering a comprehensive synthesis of available treatment modalities while emphasizing the importance of an accurate etiological diagnosis in guiding treatment planning. Unlike previous reviews, which often focus on isolated interventions or general approaches, this review highlights the necessity of personalized, etiology-specific treatment strategies. By consolidating data from a broad range of studies, it presents a structured, evidence-based approach that supports clinicians in making informed, individualized treatment decisions, ultimately enhancing both aesthetic and functional outcomes in the management of gummy smile.

## 2. Materials and Methods

### 2.1. Protocol and Registration

This systematic review was conducted according to Preferred Reporting Items for Systematic Reviews and Meta-Analyses (PRISMA), and the protocol was registered at PROSPERO under the ID CRD605156 [68].

### 2.2. Search Processing

A search on PubMed, Scopus, Web of Science, Cochrane, and Embase was performed to find papers dating from 1 January 1982 to 4 November 2024. The search strategy used the Boolean keywords ((((gummy) OR (gingiva)) AND (smile)) OR ((excessive) AND (gingival) AND (display))) AND ((orthodontic treatment) OR (orthognathic surgery)) (Table 1).

### 2.3. Inclusion Criteria

Reviewers performed a thorough evaluation of all eligible trials based on the following inclusion and exclusion criteria. Inclusion criteria: (1) Randomized Controlled Trial or Randomized Controlled Clinical Trial; (2) Studies involving human participants; (3) Studies that investigate orthodontic approaches for the correction of a gummy smile.

Studies were selected based on their relevance to orthodontic or combined orthodontic–surgical treatments for correcting excessive gingival display (gummy smile). The primary focus was on evaluating the efficacy of these interventions in improving smile aesthetics. Follow-up data were considered when available, though not required for inclusion.

### 2.4. PICOS

To systematically assess the efficacy of interventions for gummy smile across diverse patient populations and conditions, this review focuses on Population, Interventions, Comparators, Outcome, and Study Design (PICOs).

- Population: Patients with a gummy smile, defined as an excessive gingival display (≥2–3 mm), caused by various etiologies, including maxillary hyperplasia, altered passive eruption, and hyperactivity of the upper lip muscles. This review includes studies with female patients over 17 years old and male patients over 18 years old;
- Interventions: Orthodontic and orthodontic–surgical treatments, including the use of fixed appliances, temporary anchorage devices (TADs), mini-screws, and orthognathic surgery (e.g., Le Fort I osteotomy), as well as minimally invasive approaches like botulinum toxin injections and lip repositioning;
- Comparators: The comparison is between different therapeutic strategies for managing gummy smile, such as orthodontic treatments alone, combined orthodontic and surgical treatments, and minimally invasive procedures;
- Outcomes: Primary outcomes include reduction in gingival display, improvement in smile aesthetics, patient satisfaction, and treatment duration. Secondary outcomes focus on the psychological impact and quality of life improvements for patients;
- Study Desing: This review includes randomized controlled trials (RCTs) and randomized controlled clinical trials (RCCTs) published between 1 January 1982 and 4 November 2024, excluding systematic reviews, editorials, case reports, in vitro studies, and animal studies.

### 2.5. Exclusion Criteria

Exclusion criteria: (1) Systematic or literature reviews; (2) Editorials; (3) Case reports with a single case; (4) In vitro studies; (5) Studies involving animals; (6) Studies with female patients under 17 years old and male patients under 18 years old. These criteria were rigorously enforced during the article selection process to ensure that the studies included met the required standards of quality and relevance.

### 2.6. Quality Assessment and Risk of Bias

The quality of the included papers was assessed by two reviewers, A.N. and A.M.C., using the ROBINS tool, which is a tool developed to assess the risk of bias in the results of studies that compare the health effects of two or more interventions. Seven points were evaluated, and each was assigned a degree of bias. A third reviewer (F.I.) was consulted in the event of a disagreement until an agreement was reached.

**Figure 2:**
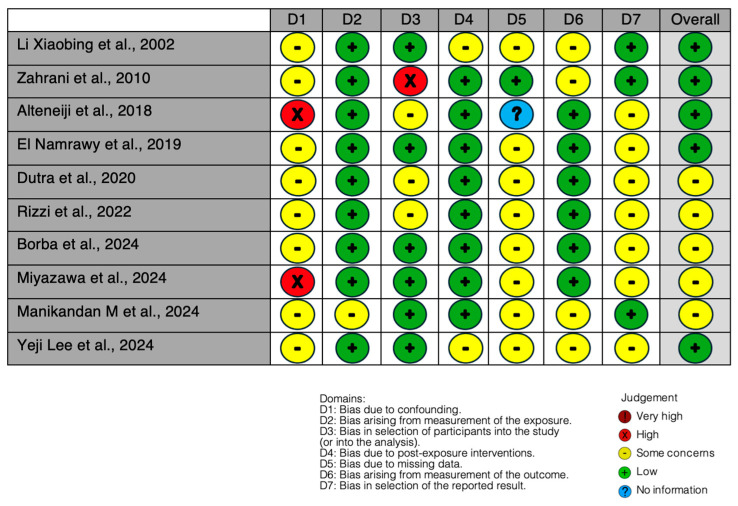
Risk of bias assessment, using Robins Tool. Li Xiaobing et al., 2002 [69]; Zahrani et al., 2010 [70]; Alteneiji et al., 2018 [71]; El Namrawy et al., 2019 [72]; Dutra et al., 2020 [73]; Rizzi et al., 2022 [74]; Borba et al., 2024 [75]; Miyazawa et al., 2024 [76]; Manikandan M et al., 2024 [77]; Yeji Lee et al., 2024 [78].

The risk of bias assessment shows moderate-to-low overall risk across the studies, with most domains rated as low risk or showing some concerns (Figure 2). Confounding and missing data present the highest concerns in specific studies, affecting the overall evaluation. Only three studies exhibit high risk in the confounding and missing data domains, while the remaining show manageable risk levels. These results indicate generally acceptable methodological quality, although careful interpretation is still required due to isolated high-risk areas.

## 3. Results

**Figure 3:**
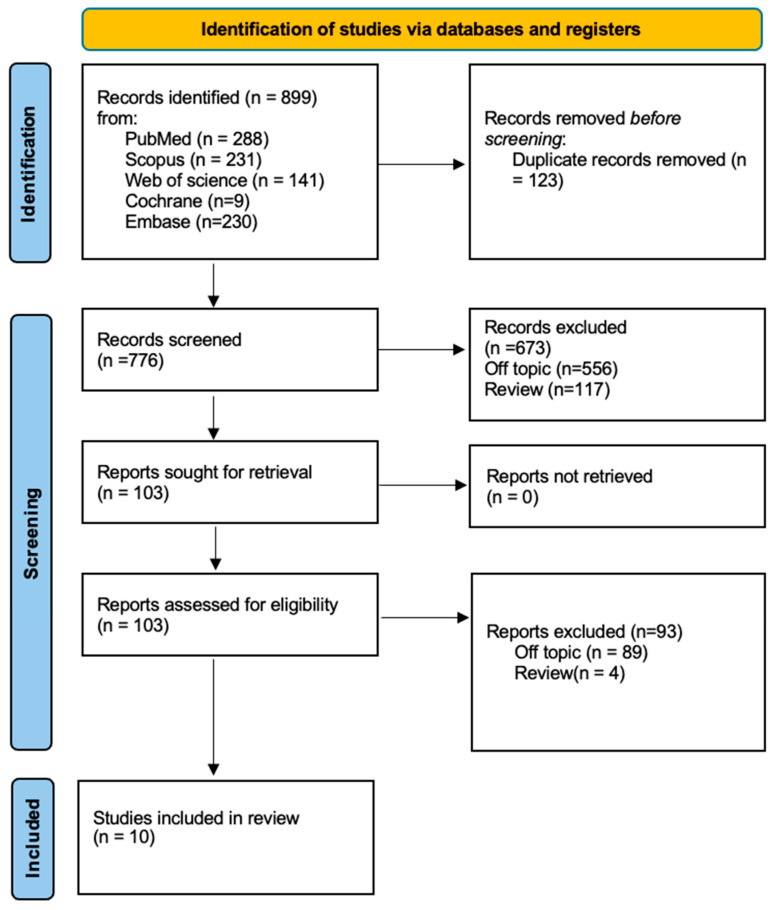
PRISMA flowchart of the inclusion process.

The database search across PubMed (288), Scopus (231), Cochrane Library (9), Web of Science (141), and Embase (230) yielded a total of 899 publications. After removing duplicates (123), 776 unique articles remained. A careful review of titles and abstracts led to the exclusion of 673 publications. The full texts of the remaining 103 articles were retrieved and assessed for eligibility. During this process, 89 articles were excluded for being irrelevant to the research topic, and 4 articles were excluded for being the reviews. Ultimately, 10 studies were included in this review for qualitative analysis (Figure 3 and Table 2). Ten studies were selected for inclusion in this review, consisting of one randomized controlled trial (RCT), one prospective clinical trial, two observational comparative studies, and six retrospective cohort studies, with a total sample size of 342 patients. The systematic review analyzes various interventions for managing gummy smiles, malocclusion, and facial aesthetic concerns across several patient populations. Alteneiji et al. (2018) [71] demonstrate that orthodontic treatment with TADs offers a non-surgical solution for vertical maxillary excess, achieving significant mandibular rotation and lip retraction. El Namrawy et al. (2019) [72] found both miniscrews and intrusive arches effective in intruding incisors, with intrusive arches slightly increasing incisor proclination. In comparison, Dutra et al. (2020) [73] and Borba et al. (2024) [75] highlight that orthognathic surgery generally provides more definitive results than botulinum toxin for reducing gingival exposure and improving smile aesthetics. Miyazawa et al. (2024) [76] showed long-term efficacy in gummy smile reduction through maxillary and mandibular molar intrusion using a modified anchorage system. Studies by Manikandan et al. (2024) [77], Li Xiaobing et al. (2002) [69], Yeji Lee et al. (2024) [78], and Zahrani et al. (2010) [70] emphasize that maxillary segmental osteotomy (AMSO) and other surgical techniques significantly enhance skeletal relationships, lip retraction, and aesthetic outcomes, particularly in Class II malocclusion cases. Across studies, surgical approaches are often more effective for severe cases, while orthodontic and non-surgical options provide viable, less invasive alternatives.

**Table 2:** Descriptive summary of item selection.

Author and Year	Study Type	Number of Patients	Patient Characteristics	Intervention	Outcome Measures	Follow-Up	Main Findings
Li Xiaobing et al. 2002 [69]	Retrospective clinical study	20	Patients with skeletal Class II, Class I, and gingival smile	Preoperative orthodontic treatment, followed by maxillary anterior segmental osteotomy (AMSO) for correction of maxillary protrusion and gingival smile.	Cephalometric measurements of soft and hard tissues, including changes in ANB angle, alveolar height, and anterior tooth protrusion.	Not specified	- Decrease in ANB angle: on average, 4.25°; - Decrease in alveolar height: about 5.5 mm for upper anterior teeth; - Decrease in protrusion of upper anterior teeth: on average, by 5 mm; - Diminution of upper and lower labial protrusion: by approximately 3.4 mm.
Zahrani et al. 2010 [70]	Observational clinical study.	20	Patients with skeletal class II. - Presented with a gingival smile, often caused by upper jaw protrusion and protrusion of the upper anterior teeth. - Treated with maxillary anterior segmental osteotomy (AMSO) in combination with orthodontic treatment.	Patients with skeletal class II. - Presented with a gingival smile - Treated with maxillary anterior segmental osteotomy (AMSO) in combination with orthodontic treatment.	Reduction in the ANB angle; reduction of anterior jaw height; reduction of the protrusion of the upper anterior teeth and the A-point; aesthetic improvement of the gingival smile.	Not specified	Significant reduction in ANB angle, anterior jaw height, and protrusion of the upper anterior teeth, improving facial aesthetics and effectively correcting the gingival smile. - The aesthetic improvement of the patient’s smile and facial profile was significant.
Alteneiji et al., 2018 [71]	Retrospective cohort study	69 patients (61 females, 8 males)	Adults (mean age 24.1) diagnosed with vertical maxillary excess (VME) and bimaxillary protrusion, gummy smile, increased mandibular plane angle, protrusive lip profile, lip incompetence, Angle Class I/II malocclusion	Orthodontic treatment with extraction therapy and extra-alveolar TADs (anterior and posterior maxillary TADs in all patients, with variations in mandibular TAD placement)	Cephalometric analysis with 24 measurements (angular and linear), evaluation of hard and soft tissue changes, lip retraction, mandibular rotation, posterior facial height reduction	Immediate post-treatment measurements, no long-term follow-up reported	Combined anterior and posterior TADs provided significant vertical control and mandibular rotation, favorable anterior teeth retraction, substantial soft tissue improvements, and non-surgical alternative for managing VME
El Namrawy et al., 2019 [72]	Prospective clinical trial	30	A total of 21 females and 9 males aged 18 to 29, with deep bite, excessive gingival display on smiling, Class I or Class II malocclusion	Group 1: maxillary incisor intrusion using miniscrews; Group 2: intrusion using an intrusive arch	Rate of intrusion, skeletal and dental effects, soft tissue changes, patient tolerance and pain	6 months	Both methods effectively intruded incisors; intrusive arch caused more upper incisor proclination; similar patient discomfort; no significant molar changes.
Dutra et al., 2020 [73]	Retrospective study	61	A total of 38 in BTX group (6 males, 32 females, mean age: 28.60 ± 6.09 years); 23 in surgery group (7 males, 16 females, mean age: 29.59 ± 5.72 years). Patients had Class I malocclusion with no anteroposterior skeletal discrepancies.	Group 1: Botulinum toxin application; Group 2: Orthognathic surgery (Le Fort I osteotomy)	Change in gingival exposure measured from extraoral photographs before and after treatment	1 month after treatment	Orthognathic surgery group showed significantly greater correction of gummy smile compared to the BTX group. Both interventions were effective, but surgery provided more definitive results.
Rizzi et al., 2022 [74]	Observational comparative study	16	Female patients, long face pattern, VME	Orthognathic surgery with Le Fort I (surg+L1) Maxillary impaction with skeletal anchorage (orthod+MP)	Visual Analog Scale (VAS) for aesthetic perception	Not specified	Significant aesthetic improvement in both groups, with OMS and orthodontists favoring surg+L1, laypeople favoring orthod+MP
Borba et al., 2024 [75]	Retrospective study	A total of 26 patients (13 treated with botulinum toxin, 13 treated with orthognathic surgery)	Group 1 (BTX): 13 patients (12 females, 1 male) with a mean age of 28.06 years (SD = 6.09) and mean gingival exposure during smiling of 5.18 mm (SD = 1.51) Group 2 (Surgical): 13 patients (9 females, 4 males) with a mean age of 30.59 years (SD = 5.72) and mean gingival exposure during smiling of 5.21 mm (SD = 1.55)	Group 1: Application of 2U of botulinum toxin into the levator labii superioris and nasalis muscles Group 2: Orthodontic treatment followed by maxillary impaction surgery (LeFort I osteotomy)	Smile attractiveness assessed using pre- and post-treatment photographs, rated by orthodontists, dentists, and laypeople on a scale of 0–10.	Group 1: Post-treatment photographs taken 14 days after botulinum toxin application Group 2: Post-treatment photographs taken shortly after orthodontic appliance removal	Both groups showed significant improvement in smile attractiveness post-treatment. However, the surgical group had significantly better final outcomes in smile attractiveness than the botulinum toxin group. Orthodontists rated the final smiles more favorably than dentists and laypeople, and orthognathic surgery was more effective in providing stable, long-term results.
Miyazawa et al., 2024 [76]	Retrospective study	16	Japanese females aged 17–33 with Class I or II malocclusion and a gummy smile of ≥3 mm gingival exposure	Midpalatal miniscrews and modified transpalatal arch (Level Anchorage System) for maxillary molar and incisor intrusion	Vertical movement of prosthion (maxillary incisor), maxillary and mandibular molar intrusion/extrusion, mandible autorotation	Mean 4 years, 2 months	Gummy smile significantly improved by intruding the maxillary first molars and incisors, resulting in upward movement of the occlusal plane and autorotation of the mandible.
Manikandan M et al. 2024 [77]	Randomized controlled trial	41	Adult subjects (mean age 30 ± 10 years) maxillary first premolar extraction, deep bite (overbite >4 mm), maxillary incisor inclination >104°, and gingival display >3 mm	- Group I: Single midline miniscrew (MS) between central incisors. - Group II: Two bilateral MS between lateral incisors and canines. - Both groups received 100 gf of force (50 gf per MS in Group II).	- Incisor intrusion and inclination - Root resorption (RR) - Alveolar bone thickness (labial and palatal) - Buccal alveolar crest height (BACH) - Sagittal changes in molar position	At 3 months post-intrusion assessment	- Intrusion: Higher in Group II (two miniscrews) - Root Resorption (RR): Higher in Group I for central incisors; higher in Group II for lateral incisors. - Incisor inclination: No significant difference between groups. - Alveolar bone changes: Significant in both groups. - Alveolar buccal ridge height: Increased in both groups. - Molar position: No significant changes.
Yeji Lee et al. 2024 [78]	Retrospective study.	44	Skeletal class II; lip protrusion; no bone metabolic diseases, craniofacial disorders, facial asymmetry > 5 mm, and a history of orthognathic surgery. Mean age of patients: 32.7 years, 13 males and 31 females.	ASO: extraction of premolars and surgical repositioning of anterior maxilla and mandible to reduce labial protrusion (In 22 cases, genioplasty).	Cephalometric Variables: ANB angle; SNB angle; lip retraction. Changes in Hard and Soft Tissues. Correction of Skeletal Class and Lip Retraction.	Evaluation of the surgical repositioning stability and aesthetic outcomes (patients satisfaction).	Effective Skeletal Correction: ASO significantly improved skeletal relationships in class II patients, reducing the ANB Significant Lip Retrusion, enhancing the facial profile. For patients with very high initial ANB and SNA values, ASO alone was not enough to achieve a normal ANB Patient Satisfaction

## 4. Discussion

The management of gummy smile in adult patients requires an interdisciplinary approach that considers the specific etiology and severity of the clinical case [25]. Therapeutic strategies can be divided into exclusively orthodontic options and combined orthodontic–surgical interventions, each with specific indications depending on the patient’s clinical characteristics [3,5].

Orthodontic techniques are the preferred choice for correcting gummy smile when the cause is dental rather than skeletal [78]. Various studies have demonstrated the effectiveness of fixed appliances and temporary anchorage devices (TADs) and miniscrews for maxillary incisor intrusion, enabling a reduction in gingival exposure without surgical intervention [71,76,77]. Manikandan et al. [77] analyzed a comparison between a single, centrally positioned miniscrew and a two-miniscrew configuration. The results indicate that the use of two miniscrews produced a more effective intrusion (2.09 ± 0.50 mm for central incisors and 2.06 ± 0.41 mm for lateral incisors), ensuring a force distribution closer to the dental arch’s center of resistance and minimizing unwanted movements [77].

Another significant study by Miyazawa et al. [76] demonstrated the effectiveness of palatal miniscrews combined with modified transpalatal arches, favoring an anterior rotation of the occlusal plane and reducing the need for invasive interventions. This approach represents a valid alternative for patients with Class I or II malocclusions, allowing for precise vertical control and improving smile aesthetics without surgical intervention [76]. Alteneiji et al. [71] explored the effectiveness of extra-alveolar TADs combined with extraction therapy to treat VME and bimaxillary protrusion, showing significant improvements in vertical maxillary control, lip retraction, and profile aesthetics [71].

In cases of gummy smile associated with more severe skeletal anomalies, such as VME, a combined orthodontic–surgical approach is often the most effective solution. This approach is indicated when skeletal discrepancies cannot be resolved solely with orthodontic techniques. Zahrani et al. [70] evaluated superior maxillary impaction via a modified Le Fort I osteotomy, which reduced maxillary vertical height by 6–8 mm, enhancing lip competence and decreasing gingival exposure. In selected cases, this procedure was combined with anterior maxillary segmental osteotomies for further reductions in protrusion, thereby improving profile aesthetics and functional occlusion [70].

In a similar study, Lee et al. [78] applied anterior segmental osteotomy (ASO) in patients with skeletal Class II to correct excessive upper lip protrusion. ASO facilitated sagittal and vertical repositioning of the anterior dentoalveolar segment, with an average incisor retraction of 6.23 mm in the maxilla and 5.66 mm in the mandible, significantly contributing to the reduction in gingival display without excessive upper lip displacement [78].

Rizzi et al. [74] highlighted the benefits of Le Fort I osteotomy in patients with severe VME, reporting stable and lasting results regarding gingival display reduction. The research compared the surgical approach with the use of miniplates for maxillary impaction, showing a slight advantage for the surgical approach in terms of long-term stability [74].

Similarly, Dutra et al. [73] demonstrated that orthognathic surgery offers a more definitive correction of gummy smile compared to botulinum toxin (BTX) injections. Although BTX significantly reduced gingival exposure, the results were temporary and not completely resolved, making this treatment a viable alternative only for patients who wished to avoid surgery or have less severe craniofacial deformities. This suggests that orthognathic surgery remains the preferred option for patients with severe skeletal misalignments, while BTX may serve as a temporary option in cases of muscle hyperactivity or for patients reluctant to undergo invasive procedures [73].

Another comparison between BTX and orthognathic surgery was conducted by Borba et al. [75], who compared the aesthetic outcomes of orthognathic surgery with botulinum toxin treatment, showing improved smile attractiveness in patients undergoing orthognathic surgery, with particularly favorable evaluations from orthodontists compared to dentists and patients themselves [75].

For deep bite correction and gingival display reduction, El Namrawy et al. [72] compared two orthodontic techniques: miniscrew-supported intrusion and the use of an intrusive arch. Both methods were effective in reducing overbite and improving the gingival display, with similar levels of incisor intrusion (2.6 mm for miniscrews and 2.9 mm for the intrusive arch). However, the intrusive arch caused more upper incisor flaring, which might have affected the overall aesthetic outcome. These findings suggest that while both techniques can successfully manage deep bite and gingival display, the choice between them should depend on the desired control over incisor inclination. Miniscrews may be more suitable for patients with proclined incisors, while the intrusive arch could be beneficial for those with retruded incisors [72].

Finally, in the study by Li et al. [69], the combined orthodontic–surgical approach involving anterior maxillary segmental osteotomy (AMSO) is evaluated for the management of gummy smile in adult patients with skeletal Class II and maxillary protrusion. This technique aims to address excessive maxillary height and protrusion, factors contributing to increased gingival display and lip incompetence. The pre-surgical orthodontic phase aligns the dental arches, establishing an optimal position for AMSO, which subsequently allows for retraction and elevation of the anterior maxillary segment. Postoperative cephalometric outcomes indicate a reduction in the ANB angle by approximately 4.25°, a decrease in anterior maxillary height by 5.5 mm, and retraction of the upper incisors by about 5 mm. These dimensional modifications significantly contribute to reducing gingival display upon smiling, directly addressing the aesthetic issues characteristic of gummy smile in this patient population [69].

The analysis of data from the included studies suggests that managing excessive gingival display in adults requires an individualized approach that considers the specific clinical features and etiology of each case. Orthodontic strategies, such as the use of temporary anchorage devices (TADs) and miniscrews for incisor intrusion, have proven effective for vertical control without surgical intervention, particularly when the etiology is predominantly dental. However, for patients with significant skeletal discrepancies, a combined orthodontic–surgical approach, including orthognathic procedures such as Le Fort I osteotomy or anterior segmental osteotomy, provides a more definitive and stable correction. The choice between purely orthodontic treatment and a combined orthodontic–surgical approach should, thus, be guided by a multidisciplinary assessment and a careful analysis of the patient’s clinical characteristics, balancing aesthetic outcomes with functional stability over the long term.

## 5. Limitations

The heterogeneity in study designs, including retrospective and prospective studies as well as observational and randomized trials, introduces potential biases and limits comparability across studies. The varying sample sizes and follow-up durations, with some studies reporting only short-term outcomes or immediate post-treatment effects, restrict the ability to assess the long-term stability and predictability of the interventions for excessive gingival display correction. Furthermore, the majority of studies included focus on specific patient demographics and clinical conditions, such as vertical maxillary excess (VME) and skeletal Class II profiles, which may not be fully generalizable to a broader population with gummy smile etiologies. The lack of standardized outcome measures across studies, particularly for aesthetic and functional evaluations, further complicates the synthesis of findings and the ability to draw consistent conclusions regarding treatment efficacy. These limitations underscore the need for more rigorous, high-quality research, with standardized methodologies and extended follow-up periods, to better evaluate the long-term effectiveness and stability of both orthodontic and orthodontic–surgical approaches.

## 6. Conclusions

This systematic review highlights the effectiveness of various orthodontic and combined orthodontic–surgical strategies in addressing gummy smile, with specific benefits depending on the etiology and severity of each case. Orthodontic methods, particularly those involving temporary anchorage devices (TADs) and miniscrews, are effective in reducing gingival display in patients without significant skeletal discrepancies. These approaches provide reliable vertical control and aesthetic improvements in the facial profile, offering non-surgical alternatives with satisfactory outcomes.

In more complex cases involving skeletal discrepancies, combined orthodontic–surgical treatments, such as maxillary impaction and anterior segmental osteotomy, have proven to be the most stable and effective options. These surgical approaches allow for substantial vertical reductions, improved lip competence, and enhanced profile aesthetics, making them suitable for managing severe skeletal misalignments that contribute to excessive gingival display. Minimally invasive treatments like botulinum toxin provide temporary reduction in gingival exposure but lack the long-term stability associated with surgical interventions.

While both orthodontic and combined strategies effectively address gummy smile, the choice of treatment should be guided by the patient’s anatomical needs and desired outcomes. The limited availability of long-term follow-up data in the reviewed studies highlights the need for further research to better assess the durability and predictability of these treatments over time, particularly for non-surgical approaches.

## Figures and Tables

**Table 1:** Database search indicators.

Database	Search Field	Results (Number of Papers Found)
PubMed	((((gummy) OR (gingiva)) AND (smile)) OR ((excessive) AND (gingival) AND (display))) AND ((orthodontic treatment) OR (orthognathic surgery))	288
Scopus	((((gummy) OR (gingiva)) AND (smile)) OR ((excessive) AND (gingival) AND (display))) AND ((orthodontic treatment) OR (orthognathic surgery))	231
Web of Science	((((gummy) OR (gingiva)) AND (smile)) OR ((excessive) AND (gingival) AND (display))) AND ((orthodontic treatment) OR (orthognathic surgery))	141
Cochrane	((((gummy) OR (gingiva)) AND (smile)) OR ((excessive) AND (gingival) AND (display))) AND ((orthodontic treatment) OR (orthognathic surgery))	9
Embase	((((gummy) OR (gingiva)) AND (smile)) OR ((excessive) AND (gingival) AND (display))) AND ((orthodontic treatment) OR (orthognathic surgery))	230

## Abbreviations

AFH	Anterior Facial Height
AMSO	Anterior Maxillary Segmental Osteotomy
ANB	Angle between Point A, Nasion, and Point B
APE	Altered Passive Eruption
ASO	Anterior Segmental Osteotomy
BACH	Buccal Alveolar Crest Height
BTX	Botulinum Toxin
CBCT	Cone-Beam Computed Tomography
CEJ	Cementoenamel Junction
EGD	Excessive Gingival Display
EMG	Electromyography
IZC	Infrazygomatic Crest
MP	Mandibular Plane
MPA	Mandibular Plane Angle
OMS	Oral and Maxillofacial Surgeons
PICOS	Population, Intervention, Comparators, Outcomes, Study Design
PRISMA	Preferred Reporting Items for Systematic Reviews and Meta-Analyses
RCT	Randomized Controlled Trial
RCCT	Randomized Controlled Clinical Trial
ROBINS	Risk Of Bias In Non-randomized Studies
S–N	Sella–Nasion
S–N/MP	Sella–Nasion to Maxillary Plane Angle
TADs	Temporary Anchorage Devices
VAS	Visual Analog Scale
VME	Vertical Maxillary Excess
